# Supplementary material for: Increased DNA methylation variability in type 1 diabetes across three immune effector cell types
Source: Nat Commun. 2016 Nov 29;7:13555. doi: 10.1038/ncomms13555 (PMC5141286; doi:10.1038/ncomms13555)
Supplement: Supplementary Information — Supplementary Figures 1-9 and Supplementary Tables 1-3. [file ncomms13555-s1.pdf]

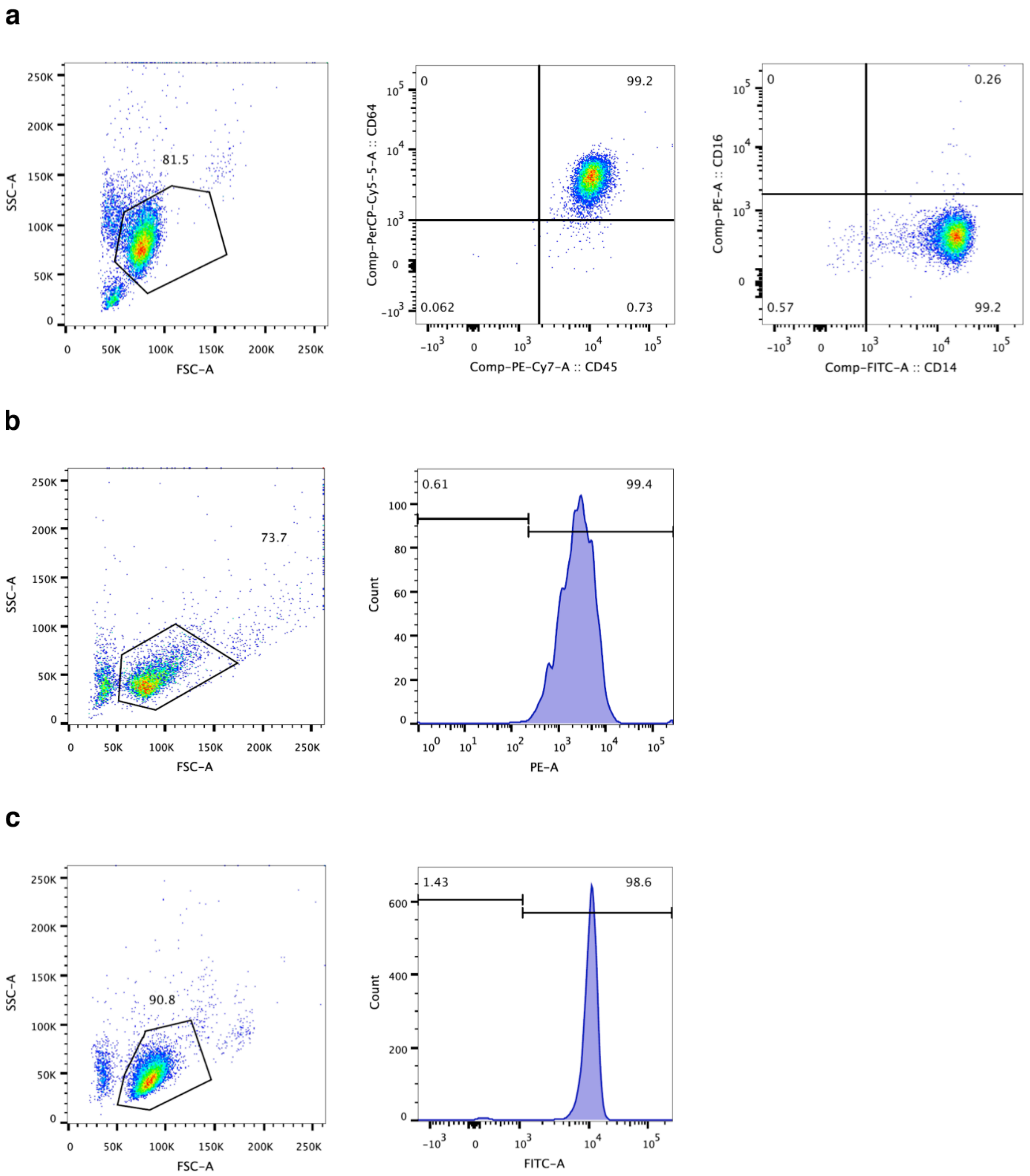

Figure 1 | Cell purity assessment using fluorescence-activated cell sorting (FACS). We show representative examples of the purity assessment in (**a**) $CD14^{+}CD16^{-}$ monocytes, (**b**) $CD19^{+}$ B cells, and (**c**) $CD4^{+}$ T cells. All cell types were purified using magnetic activated cell sorting (MACS). From each MACS-enriched cell type, we prepared two aliquots of $1{\times}10^{5}$ cells, and stained with either mouse IgG as a negative control (left) or cell type-specific antibody (right). Percentage purity was determined by FACS analysis on gated P2. In addition, we estimated cell purity directly from the DNA methylation profiles. Specifically, we assessed the relative proportions of all major leukocytes using DNA methylation signatures of an external validation set consisting of purified leukocytes (E. A. Houseman *et al.* (2012) *BMC Bioinformatics* **13**, 86). The correlation between the cell purity values assessed using FACS and the *in silico* estimate was $R = 0.66$ ($P < 2.2{\times}10^{-16}$).

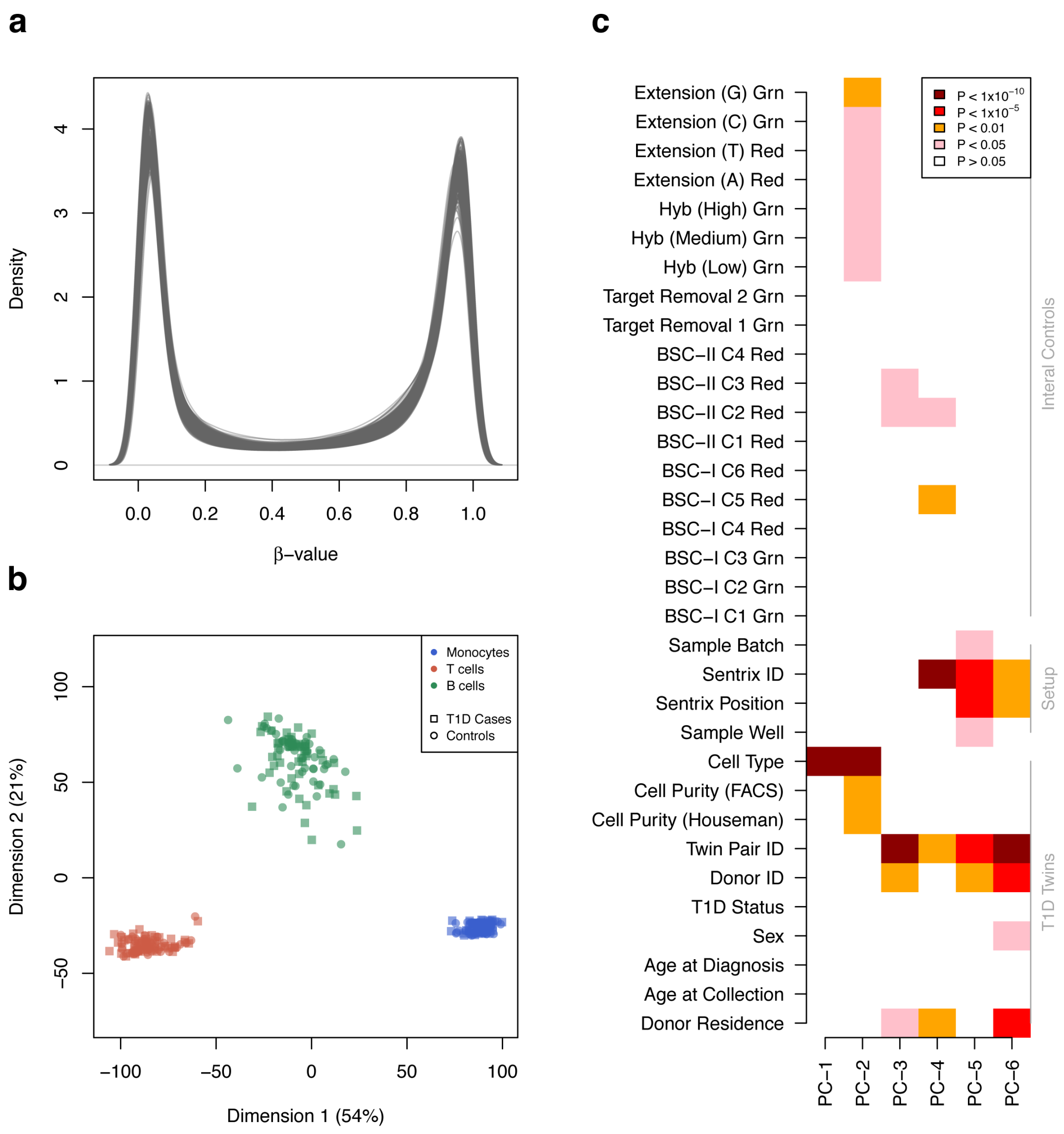

Figure 2 | Quality assessment of the Illumina Infinium HumanMethylation450 assay. The quality of the 450K array data was evaluated after normalization, probe filtering, and batch correction. (**a**) Density of DNA methylation β-values. (**b**) Multidimensional scaling indicates the similarities and differences of samples by calculating the Euclidean distances between samples based on all CpG sites, and then projecting these distances into 2D coordinates. We found Dimension 1 to associate with cell type identity, accounting for 54% of the total variance. (**c**) Singular value decomposition determines the nature of the largest components of variation (A. E. Teschendorff *et al.* (2009) *PLoS ONE* **4**, e8274). We assessed the first six principal components (PCs), and correlated these to phenotypic factors of twins (e.g. sex, age of diagnosis, and age of collection), factors related to the experimental setup (e.g. Sentrix ID, sample plate, and well), as well as internal control parameters (e.g. bisulfite conversion efficiency). We found PC1 and PC2 to significantly correlate with cell type identity ($P < 1\times10^{-10}$) and cell purity ($P < 1\times10^{-5}$), respectively. Of note, no principal component was found to correlate with T1D status.

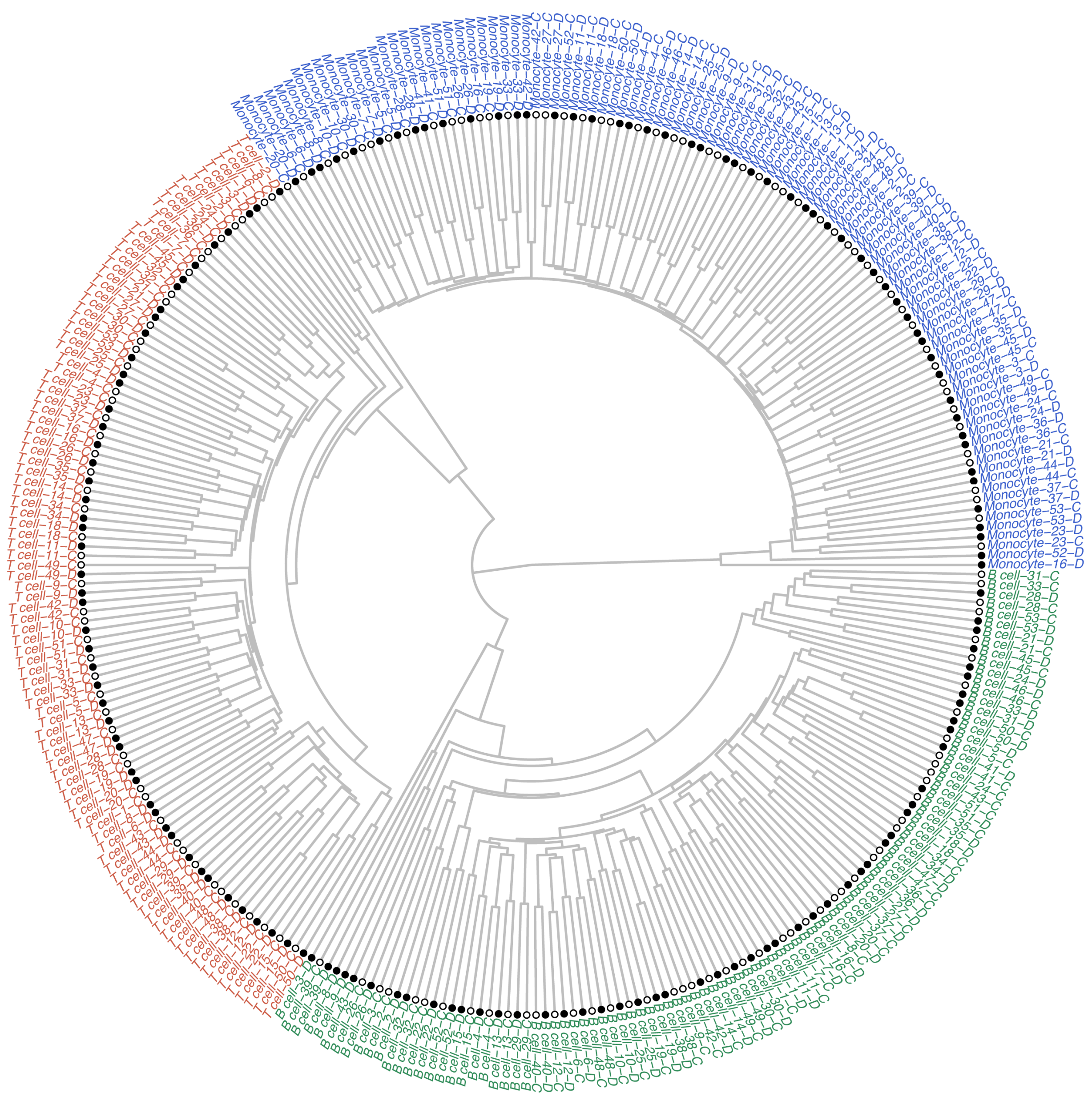

Figure 3 | Phylogenetic tree showing the relationship of DNA methylation profiles of T1D-discordant MZ twins. We constructed Euclidean distance matrices using scaled DNA methylation values. Then, hierarchical clustering of the distance matrices (method = average) was performed, and the resulting hierarchical tree visualized as phylogenetic tree using the R package ape (E. Paradis *et al.* (2004) *Bioinformatics* **20**, 289-290). The tree shows distinct clustering of the DNA methylation values according to cell type. In almost all instances, the MZ twin pairs clustered together. Note that probes containing common sequence variants were excluded from the data set. Disease status of T1D-discordant twins is indicated with a black (T1D) and white (healthy) colored tip.

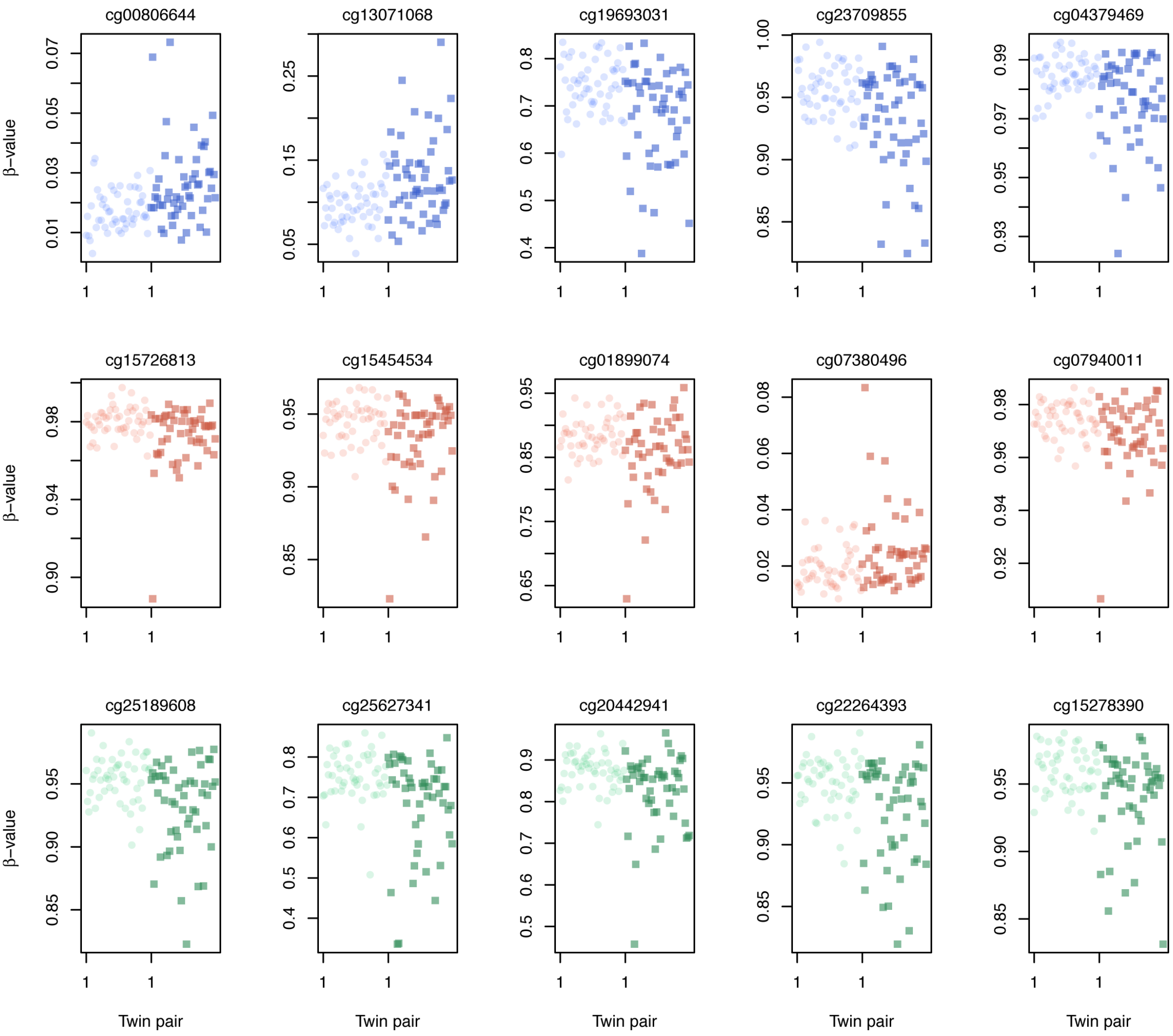

Figure 4 | Examples of T1D-associated DVPs across immune effector cell types. We show the five top-ranked T1D-associated DVPs in monocytes (top row), T cells (middle row), and B cells (bottom row). DVPs are shown that were found to be hypervariable in T1D twins (squared symbols, darker color) compared to their healthy co-twins (round symbols, lighter color) using the algorithm iEVORA. The identified DVPs represent stochastic outlier events that often occur in individual twin pairs and cell types. Samples were ordered from left to right according to disease status, and then according to twin pairing. Note that only the first twin pair is labeled.

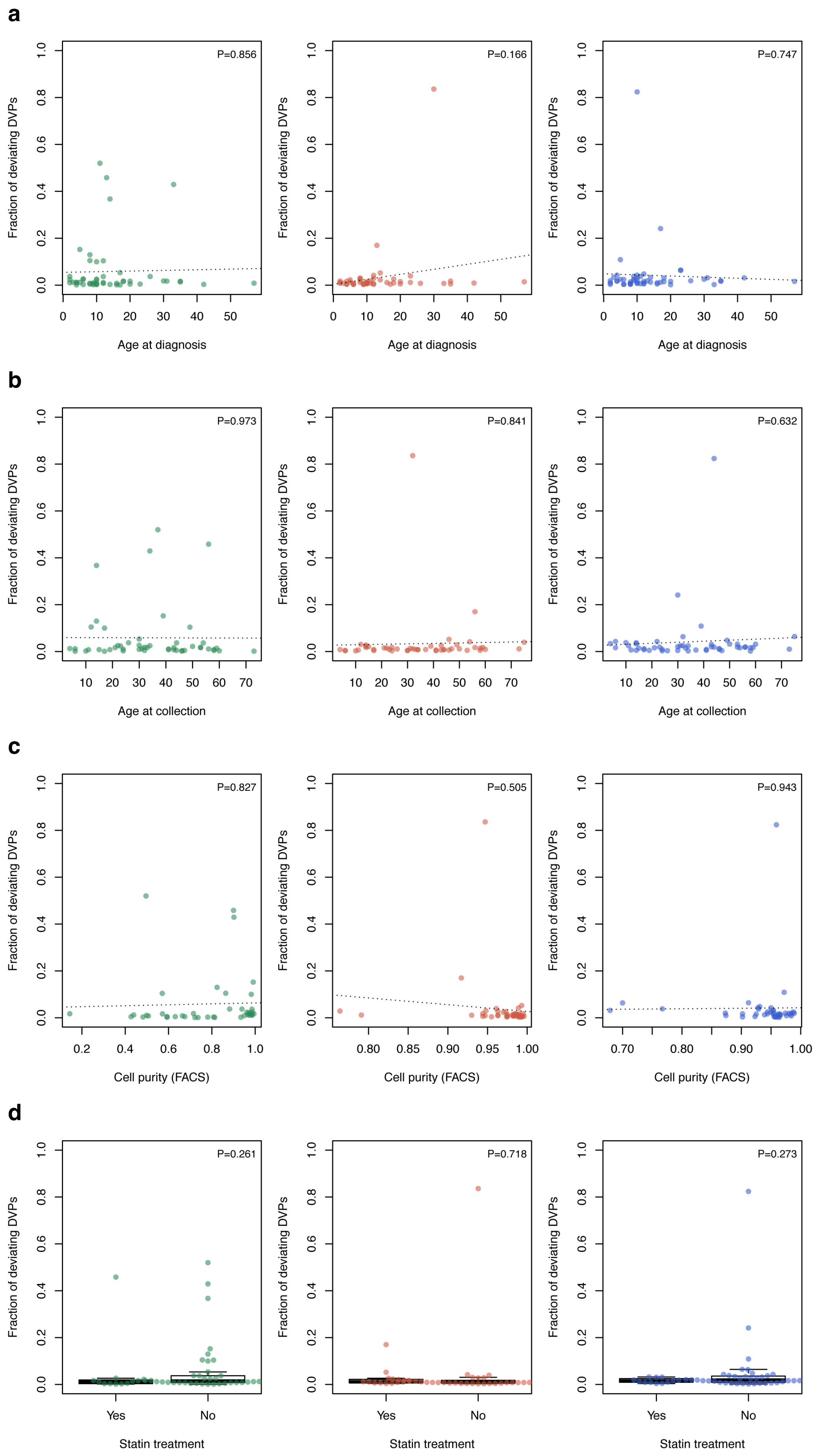
a
P=0.856
P=0.166
P=0.747
Fraction of deviating DVPs
Age at diagnosis
b
P=0.973
P=0.841
P=0.632
Age at collection
c
P=0.827
P=0.505
P=0.943
Cell purity (FACS)
d
P=0.261
P=0.718
P=0.273
Yes
No
Statin treatment

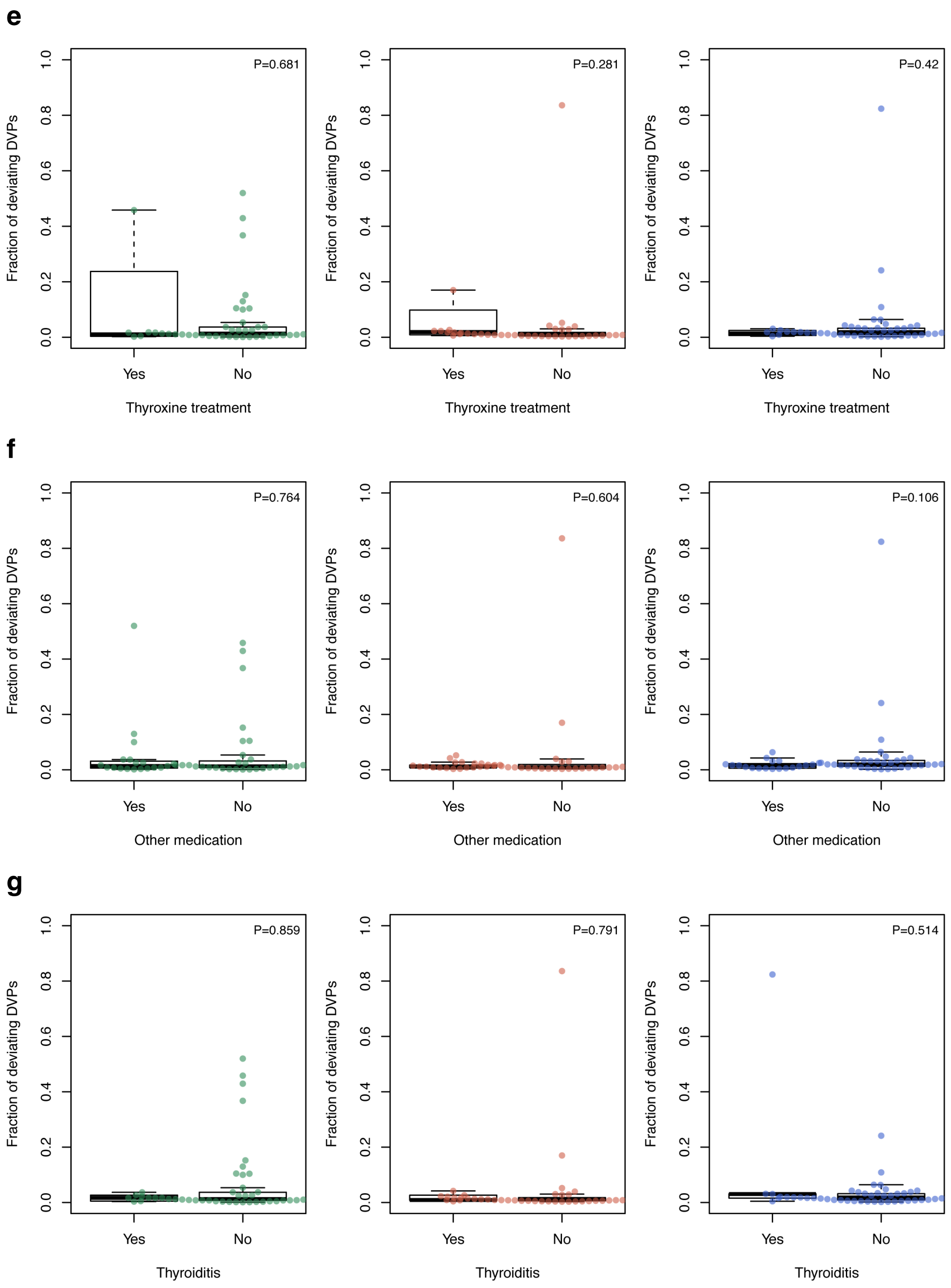

Figure 5 | Assessment of potential confounding factors for DVP discovery. For each cell type, we selected the DVPs that were found to be hypervariable in T1D twins (FDR <0.001). We then estimated the mean and standard deviation (SD) in DNA methylation across the healthy twins. We used these estimates of mean and SD to normalize the β-valued data matrix for all samples using a z-score. To estimate deviations from the healthy co-twins, one-tailed *P*-values for each T1D-associated DVP were calculated. Statistically significant deviations were defined as $P < 0.001$. Finally, the fraction of DVPs in T1D twins exhibiting a significant deviation from the healthy co-twins was correlated with potential confounding variables: (**a**) age of T1D twins at disease diagnosis; (**b**) age of T1D twins at sample collection; (**c**) cell purity of samples as quantified by FACS; (**d**) statin treatment in T1D twins;

(**e**) thyroxine treatment in T1D twins; (**f**) other medication use; (**g**) presence of thyroiditis as characterized by thyroid peroxidase autoantibodies. The three columns correspond to the cell types: B cells (left; green data points), T cells (middle; red), and monocytes (right; blue).

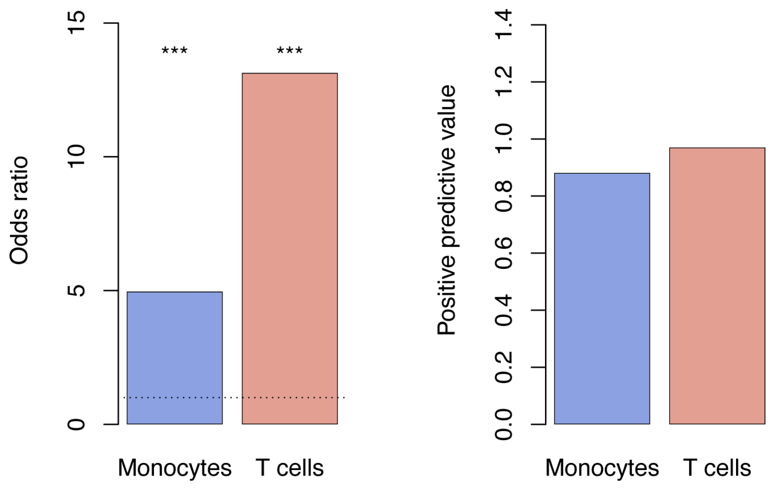

Figure 6 | Evaluation of T1D-associated DVPs contrasted to individuals with limited genetic T1D risk. Assessment of T1D-associated DVPs (FDR <0.001) in an independent 450K array data set consisting of $CD14^+$ and $CD4^+$ cells derived from 201 and 139 unrelated, healthy individuals, respectively. Bar plots showing the odds ratios of the assessment of DVPs in the independent data set (left). Stars denote statistical significance assessed using a one-tailed Fisher's exact test, i.e. $^{***}P < 1\times10^{-10}$. Positive predictive values for the analyses shown in the left panel are shown on the right panel.

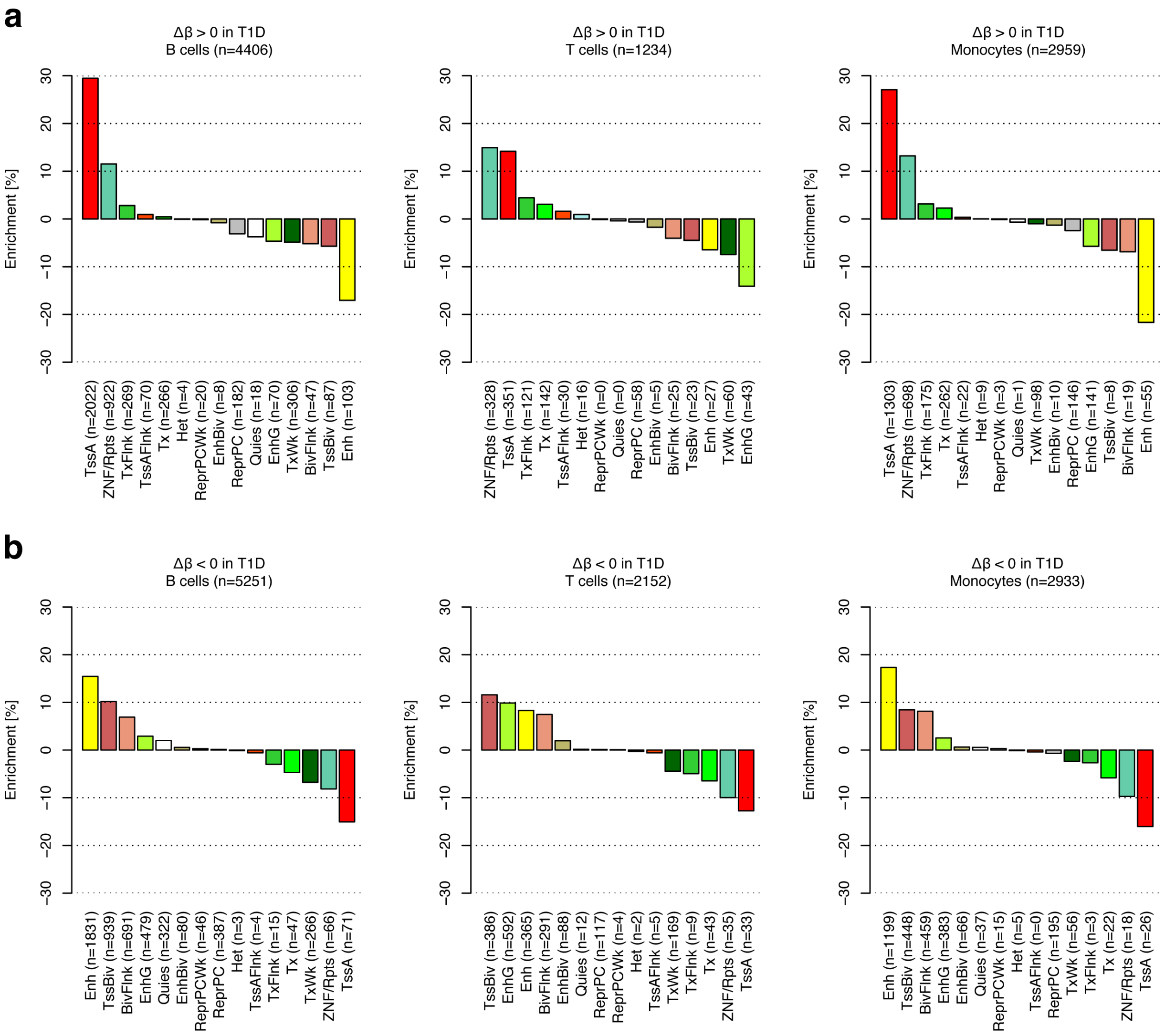

Figure 7 | Enrichment of T1D-associated DVPs at reference chromatin states. (**a**) Enrichment of DVPs at which the DNA methylation level is increased (hypermethylated; Δβ >0) in T1D twins compared to their healthy co-twins, at 15 distinct chromatin states. We selected epigenomes generated in primary cells derived from peripheral blood that correspond to the cell types investigated here. The enrichment is calculated in relation to all 450K array probes that passed quality control. (**b**) The same analyses as shown in panel (a), but for DVPs at which the DNA methylation level was reduced (hypomethylated; Δβ <0) in T1D twins. Abbreviations: TssA=active TSS; TssAFlnk=flanking active TSS; TxFlnk=transcription at 5' and 3'-end; Tx=strong transcription; TxWk=weak transcription; EnhG=genic enhancers; Enh=enhancers; ZNF/Rpts=ZNF genes and repeats; Het=heterochromatin; TssBiv=bivalent/poised TSS; BivFlnk=flanking bivalent TSS/enhancer; EnhBiv=bivalent enhancer; ReprPC=repressed Polycomb; ReprPCWk=weak repressed Polycomb; Quies=quiescent/low.

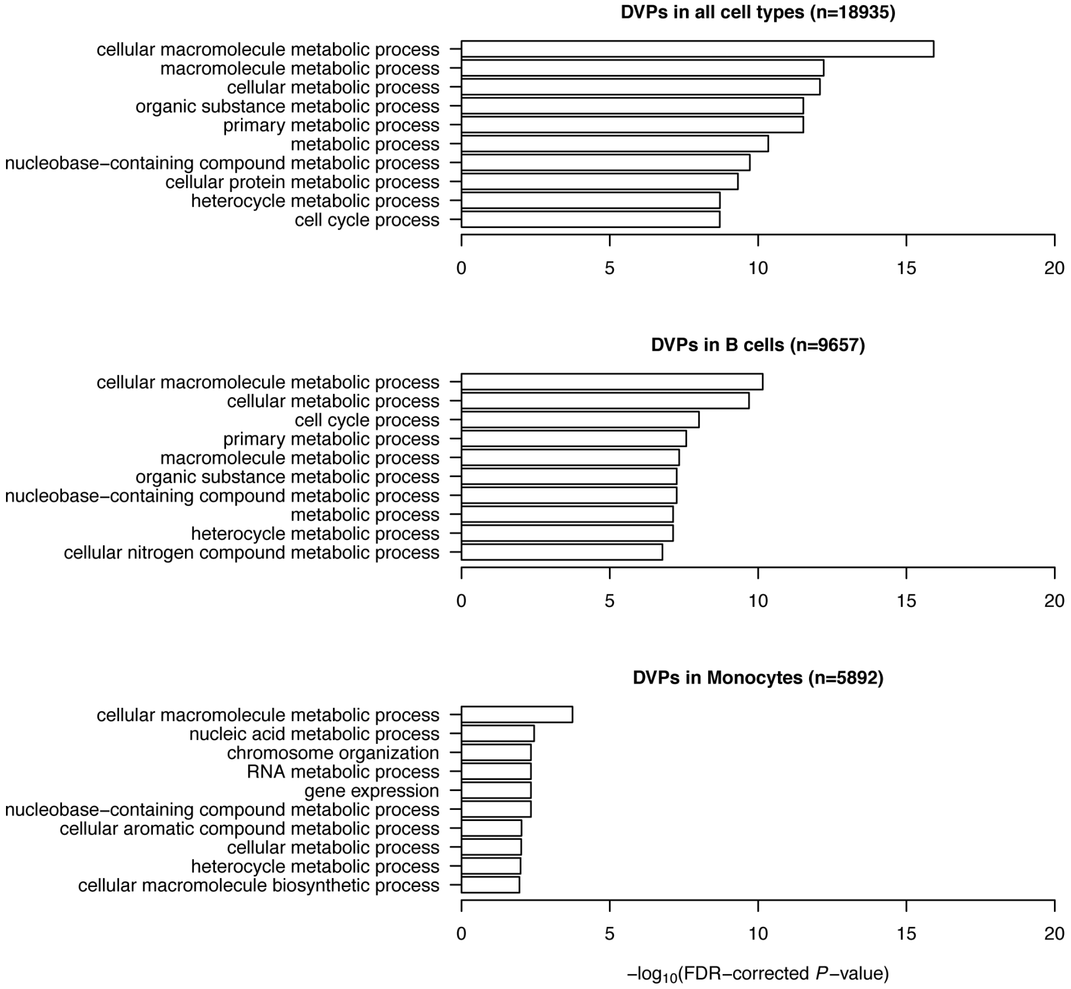

Figure 8 | Enrichment of biological process ontology terms attributed to genes in proximity to T1D-associated DVPs. We show the top ten gene ontology (GO) terms for the T1D-associated DVPs identified in all three immune cell types (top panel), B cells (middle panel), and monocytes (bottom panel). This analysis revealed enrichment of genes related to metabolic processes. $CD19^+$ B cells were mainly responsible for the observed enrichment, whereas $CD4^+$ T cells did not show a statistically significant enrichment (data not shown). This cell type-dependent enrichment pattern is plausible, as functional evidence *in vivo* suggests that naïve $CD4^+$ T cells are long-lived, with an increased cellular life span but reduced metabolic rate with age (H. Tsukamoto *et al.* (2009) *Proc. Natl. Acad. Sci. USA* **106**, 18333-18338).

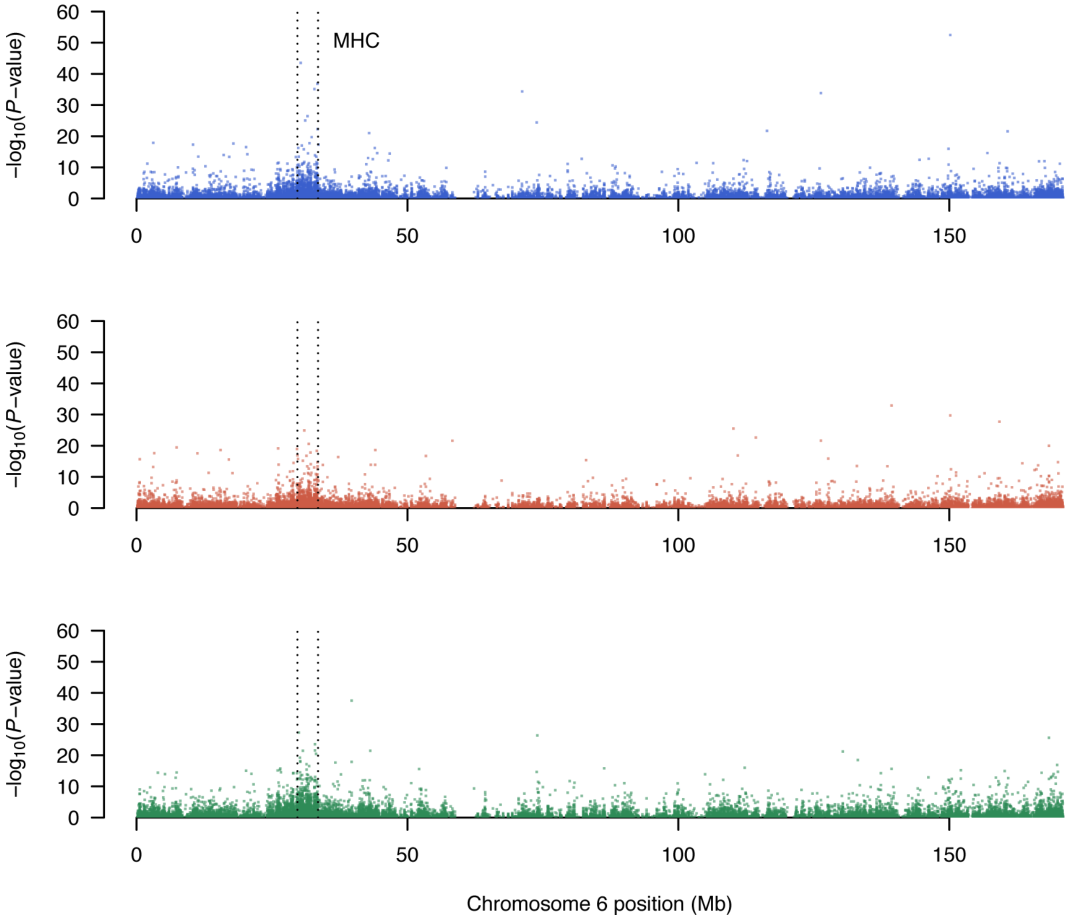

Figure 9 | Manhattan plot of DVPs at chromosome 6. We show DVPs identified in monocytes (top row), T cells (middle row), and B cells (bottom row) at chromosome 6. The MHC locus, which is key in conferring genetic risk of T1D and other autoimmune diseases, is indicated (chr6: 29,689,999–33,498,585, hg19; T1DBase v4.19). Statistical enrichment analysis of T1D-associated DVPs (FDR <0.001) mapping to the MHC locus did not reveal significance compared to all assessed CpG sites in any of the three immune cell types (P >0.05, hypergeometric test). The plot was generated using the R package qqman.

Table 1 | Sequencing statistics of WGBS-seq samples. We measured DNA methylation levels in CD4^{+} T cells in four T1D-discordant MZ twin pairs.

Barcode	Donor ID	Sample ID	Twin ID	Sex	Status	Total number of sequenced reads	Total number of mapped reads	Total number of duplicate reads	% Mapped reads	% Duplicate reads
P579	5.1	BP2	17	Male	T1D	571,076,000	542,190,000	2,945,000	94.94%	0.516%
P580	5.2	BP4	17	Male	Healthy	658,552,000	625,052,000	4,083,000	94.91%	0.620%
P581	145.1	BP6	5	Female	T1D	710,824,000	675,341,000	3,936,000	95.01%	0.554%
P582	145.2	BP8	5	Female	Healthy	612,280,000	582,577,000	3,072,000	95.15%	0.502%
P583	299.1	BP14	14	Male	T1D	655,420,000	628,097,000	3,990,000	95.83%	0.609%
P584	299.2	BP16	14	Male	Healthy	662,100,000	624,795,000	4,632,000	94.37%	0.700%
P585	329.1	BP22	4	Female	T1D	639,158,000	607,164,000	4,414,000	94.99%	0.691%
P586	329.2	BP24	4	Female	Healthy	592,602,000	562,687,000	3,226,000	94.95%	0.544%

Table 2 | Functional enrichment analyses of gene regulatory modules. The integration of T1D-associated DVPs with gene regulatory circuits in CD19[+] B cells revealed three network modules (Fig. 4f). These modules were further characterized using gene set analyses. We show enrichment of GO molecular function terms at an FDR of <0.25, which were obtained using the R packages GOstats (S. Falcon *et al.* (2007) *Bioinformatics* **23**, 257-258) and ReactomePA (G. Yu *et al.* (2016) *Mol. BioSyst.* **12**, 477-479).

Module 1:

ID	Term	Expected count	Observed count	Size	*P*-value	FDR-corrected *P*-value
GO:0015152	glucose-6-phosphate transmembrane transporter activity	0.0060	1	1	0.0060	0.1440
GO:0016263	glycoprotein-N-acetylgalactosamine 3-beta-galactosyltransferase activity	0.0060	1	1	0.0060	0.1440
GO:0019135	deoxyhypusine monooxygenase activity	0.0060	1	1	0.0060	0.1440
GO:0030229	very-low-density lipoprotein particle receptor activity	0.0060	1	1	0.0060	0.1440
GO:0034458	3'-5' RNA helicase activity	0.0060	1	1	0.0060	0.1440
GO:0090409	malonyl-CoA synthetase activity	0.0060	1	1	0.0060	0.1440
GO:0008017	microtubule binding	0.7978	4	134	0.0082	0.1440
GO:0071813	lipoprotein particle binding	0.0117	1	2	0.0117	0.1440
GO:0001156	TFIIIC-class transcription factor binding	0.0119	1	2	0.0119	0.1440
GO:0003846	2-acylglycerol O-acyltransferase activity	0.0119	1	2	0.0119	0.1440
GO:0004144	diacylglycerol O-acyltransferase activity	0.0119	1	2	0.0119	0.1440
GO:0004174	electron-transferring-flavoprotein dehydrogenase activity	0.0119	1	2	0.0119	0.1440
GO:0004372	glycine hydroxymethyltransferase activity	0.0119	1	2	0.0119	0.1440
GO:0008732	L-allo-threonine aldolase activity	0.0119	1	2	0.0119	0.1440
GO:0042610	CD8 receptor binding	0.0119	1	2	0.0119	0.1440
GO:0050252	retinol O-fatty-acyltransferase activity	0.0119	1	2	0.0119	0.1440
GO:0003682	chromatin binding	1.9618	6	334	0.0131	0.1500
GO:0003714	transcription corepressor activity	0.9883	4	166	0.0169	0.1640
GO:0001030	RNA polymerase III type 1 promoter DNA binding	0.0179	1	3	0.0178	0.1640
GO:0001031	RNA polymerase III type 2 promoter DNA binding	0.0179	1	3	0.0178	0.1640
GO:0001032	RNA polymerase III type 3 promoter DNA binding	0.0179	1	3	0.0178	0.1640
GO:0032050	clathrin heavy chain binding	0.0238	1	4	0.0236	0.1908
GO:0042609	CD4 receptor binding	0.0238	1	4	0.0236	0.1908
GO:0046976	histone methyltransferase activity (H3-K27 specific)	0.0238	1	4	0.0236	0.1908
GO:0046974	histone methyltransferase activity (H3-K9 specific)	0.0298	1	5	0.0294	0.2191
GO:0097027	ubiquitin-protein transferase activator activity	0.0298	1	5	0.0294	0.2191
GO:0051117	ATPase binding	0.2739	2	46	0.0305	0.2191
GO:0001948	glycoprotein binding	0.2858	2	48	0.0330	0.2203
GO:0000182	rDNA binding	0.0357	1	6	0.0352	0.2203
GO:0005041	low-density lipoprotein receptor activity	0.0357	1	6	0.0352	0.2203
GO:0016832	aldehyde-lyase activity	0.0357	1	6	0.0352	0.2203
GO:0034185	apolipoprotein binding	0.0417	1	7	0.0410	0.2483

Module 2:

ID	Term	Expected count	Observed count	Size	*P*-value	FDR-corrected *P*-value
GO:0017025	TBP-class protein binding	0.0971	2	15	0.0041	0.1384
GO:0003867	4-aminobutyrate transaminase activity	0.0065	1	1	0.0065	0.1384
GO:0032145	succinate-semialdehyde dehydrogenase binding	0.0065	1	1	0.0065	0.1384
GO:0045352	interleukin-1 Type I receptor antagonist activity	0.0065	1	1	0.0065	0.1384

GO:0045353	interleukin-1 Type II receptor antagonist activity	0.0065	1	1	0.0065	0.1384
GO:0047298	(S)-3-amino-2-methylpropionate transaminase activity	0.0065	1	1	0.0065	0.1384
GO:0017112	Rab guanyl-nucleotide exchange factor activity	0.1489	2	23	0.0096	0.1384
GO:0001104	RNA polymerase II transcription cofactor activity	0.4468	3	69	0.0100	0.1384
GO:0004485	methylcrotonoyl-CoA carboxylase activity	0.0130	1	2	0.0129	0.1384
GO:0005150	interleukin-1, Type I receptor binding	0.0130	1	2	0.0129	0.1384
GO:0005151	interleukin-1, Type II receptor binding	0.0130	1	2	0.0129	0.1384
GO:0005280	hydrogen:amino acid symporter activity	0.0130	1	2	0.0129	0.1384
GO:0015187	glycine transmembrane transporter activity	0.0130	1	2	0.0129	0.1384
GO:0016422	mRNA (2'-O-methyladenosine-N6-)-methyltransferase activity	0.0130	1	2	0.0129	0.1384
GO:0019911	structural constituent of myelin sheath	0.0130	1	2	0.0129	0.1384
GO:0045505	dynein intermediate chain binding	0.0130	1	2	0.0129	0.1384
GO:0050682	AF-2 domain binding	0.0130	1	2	0.0129	0.1384
GO:0051747	cytosine C-5 DNA demethylase activity	0.0130	1	2	0.0129	0.1384
GO:0008173	RNA methyltransferase activity	0.1878	2	29	0.0150	0.1433
GO:0003726	double-stranded RNA adenosine deaminase activity	0.0194	1	3	0.0193	0.1433
GO:0004582	dolichyl-phosphate beta-D-mannosyltransferase activity	0.0194	1	3	0.0193	0.1433
GO:0005049	nuclear export signal receptor activity	0.0194	1	3	0.0193	0.1433
GO:0015093	ferrous iron transmembrane transporter activity	0.0194	1	3	0.0193	0.1433
GO:0015193	L-proline transmembrane transporter activity	0.0194	1	3	0.0193	0.1433
GO:0043734	DNA-N1-methyladenine dioxygenase activity	0.0194	1	3	0.0193	0.1433
GO:0044020	histone methyltransferase activity (H4-R3 specific)	0.0194	1	3	0.0193	0.1433
GO:0004075	biotin carboxylase activity	0.0259	1	4	0.0257	0.1547
GO:0009374	biotin binding	0.0259	1	4	0.0257	0.1547
GO:0015180	L-alanine transmembrane transporter activity	0.0259	1	4	0.0257	0.1547
GO:0016494	C-X-C chemokine receptor activity	0.0259	1	4	0.0257	0.1547
GO:0019966	interleukin-1 binding	0.0259	1	4	0.0257	0.1547
GO:0048273	mitogen-activated protein kinase p38 binding	0.0259	1	4	0.0257	0.1547
GO:0030374	ligand-dependent nuclear receptor transcription coactivator activity	0.2720	2	42	0.0301	0.1760
GO:0003725	double-stranded RNA binding	0.2914	2	45	0.0342	0.1800
GO:0004169	dolichyl-phosphate-mannose-protein mannosyltransferase activity	0.0389	1	6	0.0382	0.1800
GO:0005104	fibroblast growth factor receptor binding	0.0389	1	6	0.0382	0.1800
GO:0005168	neurotrophin TRKA receptor binding	0.0389	1	6	0.0382	0.1800
GO:0016885	ligase activity, forming carbon-carbon bonds	0.0389	1	6	0.0382	0.1800
GO:0034452	dynactin binding	0.0389	1	6	0.0382	0.1800
GO:0035242	protein-arginine omega-N asymmetric methyltransferase activity	0.0389	1	6	0.0382	0.1800
GO:0036402	proteasome-activating ATPase activity	0.0389	1	6	0.0382	0.1800
GO:0005165	neurotrophin receptor binding	0.0453	1	7	0.0445	0.1951
GO:0042813	Wnt-activated receptor activity	0.0453	1	7	0.0445	0.1951
GO:0048019	receptor antagonist activity	0.0453	1	7	0.0445	0.1951
GO:0005085	guanyl-nucleotide exchange factor activity	0.8030	3	124	0.0462	0.1980
GO:0004860	protein kinase inhibitor activity	0.3497	2	54	0.0476	0.1996
GO:0030545	receptor regulator activity	0.0510	1	8	0.0499	0.1996
GO:0005537	mannose binding	0.0518	1	8	0.0507	0.1996
GO:0016273	arginine N-methyltransferase activity	0.0518	1	8	0.0507	0.1996
GO:0005068	transmembrane receptor protein tyrosine kinase adaptor activity	0.0583	1	9	0.0568	0.2109
GO:0005072	transforming growth factor beta receptor, cytoplasmic mediator activity	0.0583	1	9	0.0568	0.2109
GO:0055106	ubiquitin-protein transferase regulator activity	0.0583	1	9	0.0568	0.2109
GO:0016741	transferase activity, transferring one-carbon groups	0.8872	3	137	0.0589	0.2131
GO:0017110	nucleoside-diphosphatase activity	0.0648	1	10	0.0629	0.2131
GO:0019211	phosphatase activator activity	0.0648	1	10	0.0629	0.2131
GO:0043422	protein kinase B binding	0.0648	1	10	0.0629	0.2131
GO:0043522	leucine zipper domain binding	0.0648	1	10	0.0629	0.2131

GO:0004861	cyclin-dependent protein serine/threonine kinase inhibitor activity	0.0712	1	11	0.0690	0.2257
GO:0043274	phospholipase binding	0.0712	1	11	0.0690	0.2257
GO:0005385	zinc ion transmembrane transporter activity	0.0777	1	12	0.0750	0.2370
GO:0043014	alpha-tubulin binding	0.0777	1	12	0.0750	0.2370
GO:0008171	O-methyltransferase activity	0.0842	1	13	0.0810	0.2370
GO:0017147	Wnt-protein binding	0.0842	1	13	0.0810	0.2370
GO:0030546	receptor activator activity	0.0842	1	13	0.0810	0.2370
GO:0031434	mitogen-activated protein kinase kinase binding	0.0842	1	13	0.0810	0.2370
GO:0031489	myosin V binding	0.0842	1	13	0.0810	0.2370
GO:0008301	DNA binding, bending	0.0907	1	14	0.0870	0.2469
GO:0016769	transferase activity, transferring nitrogenous groups	0.0907	1	14	0.0870	0.2469

**Module 3:**						
ID	Term	Expected count	Observed count	Size	*P*-value	FDR-corrected *P*-value
GO:0001105	RNA polymerase II transcription coactivator activity	0.3943	4	25	0.0006	0.1976
GO:0035064	methylated histone binding	0.5362	4	34	0.0019	0.2027
GO:0046790	virion binding	0.0946	2	6	0.0036	0.2027
GO:0098811	transcriptional repressor activity, RNA polymerase II activating transcription factor binding	0.6466	4	41	0.0038	0.2027
GO:0016407	acetyltransferase activity	1.0725	5	68	0.0043	0.2027
GO:0008374	O-acyltransferase activity	0.3628	3	23	0.0054	0.2027
GO:0003841	1-acylglycerol-3-phosphate O-acyltransferase activity	0.1419	2	9	0.0083	0.2027
GO:0016746	transferase activity, transferring acyl groups	2.3658	7	150	0.0095	0.2027
GO:0071617	lysophospholipid acyltransferase activity	0.1735	2	11	0.0124	0.2027
GO:0016410	N-acyltransferase activity	0.9148	4	58	0.0131	0.2027
GO:0001223	transcription coactivator binding	0.0158	1	1	0.0158	0.2027
GO:0003854	3-beta-hydroxy-delta5-steroid dehydrogenase activity	0.0158	1	1	0.0158	0.2027
GO:0004092	carnitine O-acetyltransferase activity	0.0158	1	1	0.0158	0.2027
GO:0004134	4-alpha-glucanotransferase activity	0.0158	1	1	0.0158	0.2027
GO:0004135	amylo-alpha-1,6-glucosidase activity	0.0158	1	1	0.0158	0.2027
GO:0004139	deoxyribose-phosphate aldolase activity	0.0158	1	1	0.0158	0.2027
GO:0004605	phosphatidate cytidylyltransferase activity	0.0158	1	1	0.0158	0.2027
GO:0004751	ribose-5-phosphate isomerase activity	0.0158	1	1	0.0158	0.2027
GO:0004945	angiotensin type II receptor activity	0.0158	1	1	0.0158	0.2027
GO:0005139	interleukin-7 receptor binding	0.0158	1	1	0.0158	0.2027
GO:0036139	peptidyl-histidine dioxygenase activity	0.0158	1	1	0.0158	0.2027
GO:0036140	peptidyl-asparagine 3-dioxygenase activity	0.0158	1	1	0.0158	0.2027
GO:0038052	RNA polymerase II transcription factor activity, estrogen-activated sequence-specific DNA binding	0.0158	1	1	0.0158	0.2027
GO:0042008	interleukin-18 receptor activity	0.0158	1	1	0.0158	0.2027
GO:0047888	fatty acid peroxidase activity	0.0158	1	1	0.0158	0.2027
GO:0050827	toxin receptor binding	0.0158	1	1	0.0158	0.2027
GO:0050681	androgen receptor binding	0.5362	3	34	0.0161	0.2027
GO:0034212	peptide N-acetyltransferase activity	0.6114	3	39	0.0229	0.2096
GO:0004402	histone acetyltransferase activity	0.6309	3	40	0.0248	0.2096
GO:0001076	transcription factor activity, RNA polymerase II transcription factor binding	1.6876	5	107	0.0269	0.2096
GO:0051015	actin filament binding	1.1513	4	73	0.0280	0.2096
GO:0001012	RNA polymerase II regulatory region DNA binding	0.0315	1	2	0.0312	0.2096
GO:0001537	N-acetylgalactosamine 4-O-sulfotransferase activity	0.0315	1	2	0.0313	0.2096
GO:0003955	NAD(P)H dehydrogenase (quinone) activity	0.0315	1	2	0.0313	0.2096
GO:0004329	formate-tetrahydrofolate ligase activity	0.0315	1	2	0.0313	0.2096
GO:0004816	asparagine-tRNA ligase activity	0.0315	1	2	0.0313	0.2096

GO:0005007	fibroblast growth factor-activated receptor activity	0.0315	1	2	0.0313	0.2096
GO:0005243	gap junction channel activity	0.0315	1	2	0.0313	0.2096
GO:0008240	tripeptidyl-peptidase activity	0.0315	1	2	0.0313	0.2096
GO:0008559	xenobiotic-transporting ATPase activity	0.0315	1	2	0.0313	0.2096
GO:0008941	nitric oxide dioxygenase activity	0.0315	1	2	0.0313	0.2096
GO:0015433	peptide antigen-transporting ATPase activity	0.0315	1	2	0.0313	0.2096
GO:0018479	benzaldehyde dehydrogenase (NAD+) activity	0.0315	1	2	0.0313	0.2096
GO:0019826	oxygen sensor activity	0.0315	1	2	0.0313	0.2096
GO:0033142	progesterone receptor binding	0.0315	1	2	0.0313	0.2096
GO:0045155	electron transporter, transferring electrons from CoQH2-cytochrome c reductase complex and cytochrome c oxidase complex activity	0.0315	1	2	0.0313	0.2096
GO:0046980	tapasin binding	0.0315	1	2	0.0313	0.2096
GO:0047756	chondroitin 4-sulfotransferase activity	0.0315	1	2	0.0313	0.2096
GO:0050659	N-acetylgalactosamine 4-sulfate 6-O-sulfotransferase activity	0.0315	1	2	0.0313	0.2096
GO:0071532	ankyrin repeat binding	0.0315	1	2	0.0313	0.2096
GO:0020037	heme binding	0.6940	3	44	0.0318	0.2096
GO:0016922	ligand-dependent nuclear receptor binding	0.2839	2	18	0.0320	0.2096
GO:0051117	ATPase binding	0.7255	3	46	0.0356	0.2286
GO:0004096	catalase activity	0.0473	1	3	0.0466	0.2474
GO:0004591	oxoglutarate dehydrogenase (succinyl-transferring) activity	0.0473	1	3	0.0466	0.2474
GO:0004748	ribonucleoside-diphosphate reductase activity, thioredoxin disulfide as acceptor	0.0473	1	3	0.0466	0.2474
GO:0005344	oxygen transporter activity	0.0473	1	3	0.0466	0.2474
GO:0016728	oxidoreductase activity, acting on CH or CH2 groups, disulfide as acceptor	0.0473	1	3	0.0466	0.2474
GO:0031698	beta-2 adrenergic receptor binding	0.0473	1	3	0.0466	0.2474
GO:0034056	estrogen response element binding	0.0473	1	3	0.0466	0.2474
GO:0043533	inositol 1,3,4,5 tetrakisphosphate binding	0.0473	1	3	0.0466	0.2474
GO:0046978	TAP1 binding	0.0473	1	3	0.0466	0.2474
GO:0050693	LBD domain binding	0.0473	1	3	0.0466	0.2474
GO:0052650	NADP-retinol dehydrogenase activity	0.0473	1	3	0.0466	0.2474

Table 3 | Further gene set analysis of network module 1. We performed additional functional enrichment analyses of module 1 (Fig. 4f and Table 2) at an FDR of <0.01 using Cytoscape (P. Shannon *et al.* (2003) *Genome Res.* **13**, 2498-2504) and ClueGO (G. Bindea *et al.* (2009) *Bioinformatics* **25**, 1091-1093).

ID	Ontology	Term	Count genes	Proportion of associated genes	Term		Group		Associated genes
					P-value	FDR-corrected *P*-value	*P*-value	FDR-corrected *P*-value	
GO:0019888	Molecular function	protein phosphatase regulator activity	3	6.12%	0.0026	0.0026	0.0026	0.0026	*MKL2, NSFL1C, PPP1R14A*
GO:0032006	Biological process	regulation of TOR signaling	3	6.25%	0.0025	0.0031	0.0025	0.0037	*FAM83D, GOLPH3, RPTOR*
GO:0055088	Biological process	lipid homeostasis	4	5.48%	0.0008	0.0038	0.0011	0.0034	*DGAT1, LDLR, NR5A2, SLC37A4*
GO:0006641	Biological process	triglyceride metabolic process	4	4.94%	0.0011	0.0018	0.0011	0.0034	*ACSF3, DGAT1, LDLR, SLC37A4*
GO:0042632	Biological process	cholesterol homeostasis	3	8.57%	0.0010	0.0025	0.0011	0.0034	*LDLR, NR5A2, SLC37A4*
